# Neuromotor recovery from stroke: computational models at central, functional, and muscle synergy level

**DOI:** 10.3389/fncom.2013.00097

**Published:** 2013-08-22

**Authors:** Maura Casadio, Irene Tamagnone, Susanna Summa, Vittorio Sanguineti

**Affiliations:** Department of Informatics, Bioengineering, Robotics and Systems Engineering, Neuroengineering and Neurorobotics Lab (NeuroLAB), University of GenoaGenoa, Italy

**Keywords:** functional recovery, cortical reorganization, motor skill learning, compensation, robot, slacking, muscle synergy

## Abstract

Computational models of neuromotor recovery after a stroke might help to unveil the underlying physiological mechanisms and might suggest how to make recovery faster and more effective. At least in principle, these models could serve: (i) To provide testable hypotheses on the nature of recovery; (ii) To predict the recovery of individual patients; (iii) To design patient-specific “optimal” therapy, by setting the treatment variables for maximizing the amount of recovery or for achieving a better generalization of the learned abilities across different tasks. Here we review the state of the art of computational models for neuromotor recovery through exercise, and their implications for treatment. We show that to properly account for the computational mechanisms of neuromotor recovery, multiple levels of description need to be taken into account. The review specifically covers models of recovery at central, functional and muscle synergy level.

## Introduction

In the nervous system, a cerebro-vascular accident (stroke) elicits a complex series of reorganization processes at molecular, cellular, neural population, behavioral (sensorimotor and cognitive) and social interaction levels, with temporal scales that range from hours, to months, to years (Schaechter, 2004; Barbay et al., 2006; Nudo, 2006, 2007). Alterations occurs well beyond the actual lesion, including a low-activity “penumbra” region in the surrounding areas and inter-hemispheric unbalance due to a decreased activity in the ipsilesional side (Hummel and Cohen, 2006).

Animal models and human studies suggest that functional recovery is mediated by use-dependent reorganization of the preserved neural circuitry. A key to neuromotor recovery, and the basis of neuro-rehabilitation interventions, is movement associated with a task (Nudo, 2006, 2007) and with volitional effort (Blennerhassett and Dite, 2004; Higgins et al., 2006; Timmermans et al., 2010). This process produces alterations in neuronal excitability (Ward and Cohen, 2004), leading to changes in neural circuitry, with a process resembling that occurring in the developing brain. Redundancy in the musculoskeletal system plays a key role in neuromotor recovery. It has long been suggested (Bernstein, 1967) that the nervous system has a modular control structure to deal with redundancy. According to this view, the nervous system adaptively controls combinations of motor primitives that are the “building blocks” of movement organization. The pressure toward re-gaining functional independence may lead to the development of compensatory strategies that, even when adequate for carrying out activities of daily life (ADLs), may be stereotypical or energetically inefficient so that they may ultimately prevent true recovery (Levin, 1996b; Cirstea and Levin, 2000). For instance, an excess use of the non-paretic limb can have a negative influence on the process of cortical reorganization (Avanzino et al., 2011) by further reinforcing the imbalance between the two hemispheres. Models of neuromotor recovery that explicitly take modularity into account might be the most appropriate level of description for these phenomena.

In summary, neuromotor recovery through exercise is the end result of a complex interplay between activity-dependent reorganization of the brain areas close to the lesion, the recruitment of new neural pathways and the development of novel motor strategies.

A deeper understanding of the functional and physiological mechanisms underlying recovery would have strong impact on approaches to neuromotor rehabilitation. Computational motor control and, more in general, computational models may greatly contribute to this understanding (Huang and Krakauer, 2009). Even more importantly, models may be directly incorporated into technological solutions, and can constitute the basis for personalized therapy. In fact, Marchal-Crespo and Reinkensmeyer (2009) pointed out that there is a specific need for “improved models of human motor recovery to provide a more rational framework for designing robotic therapy control strategies.” However, while musculoskeletal models have a long history in the personalization of treatment of movement disorders (Fregly et al., 2012), computational models of neuromotor recovery through exercise are still in their infancy.

Here, we review the state of the art of computational models for neuromotor recovery and their implications for treatment. We then suggest directions for future research.

## Models of neuromotor recovery

There have been several attempts to model the time course of recovery, either when it is spontaneous, or when is facilitated by some form of treatment, e.g., electrical stimulation or assistance by a robot. Here we specifically focus on models of activity-dependent recovery. Models of recovery may focus on different levels of description, ranging from cortical or subcortical lesions, to muscle control, to functional behavior in the context of a specific task.

Models of neuromotor recovery at the level of cortical circuitry (Goodall et al., 1997; Reinkensmeyer et al., 2003; Butz et al., 2009) address how focal cortical lesions elicit neural reorganization phenomena, and the way these lesions affect motor behavior.

Other models address the “functional” level of description, related to the ability to complete a specific task and to how it changes over time. For instance, Colombo et al. (2008, 2010) describe the temporal evolution of performance over training time. Only few models focus on how physical interaction affects voluntary control (Emken et al., 2007), and how such voluntary control changes with exercise (Casadio and Sanguineti, 2012).

Models of muscle control focus on characterizing the impairment in individual subjects in terms of altered muscle synergies (Cheung et al., 2012).

Only few models encompass multiple levels of description. Han et al. (2008), Reinkensmeyer et al. (2012a) and Takiyama and Okada (2012) address the mechanisms of cortical reorganization in the context of voluntary motor activity, in a skill learning scenario. The emphasis here is on how voluntary movements promote recovery through cortical and subcortical reorganization.

In the following sections we will review a number of computational models of neuromotor recovery—respectively, at central, functional and muscle level—that have recently appeared in the literature. For each model we provide a general description; we then discuss their main findings or predictions, their limitations and the implications for rehabilitation.

### Models of focal cortical lesions and activity-dependent reorganization

Several models of neuromotor recovery explicitly focus on the mechanisms of cortical reorganization following a focal lesion (models at central level).

**Figure 1:**
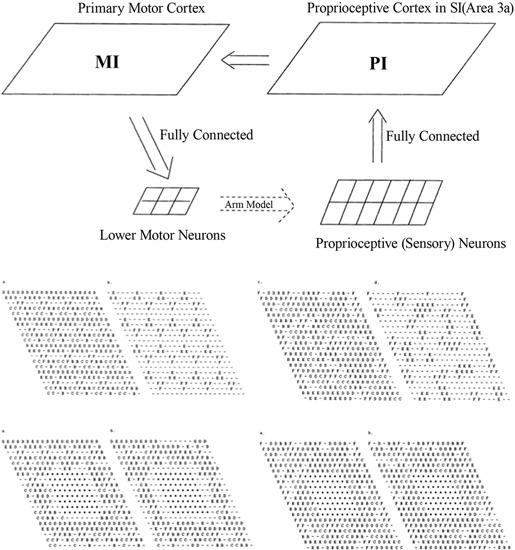
**Top: Sensorimotor control model, involving the primary motor cortex (MI) and the proprioceptive cortex (PI)**. Middle: Intact PI (left) and MI (right) maps. Letters indicate, for each neuron, the muscle groups of which that neuron encodes the activity (or stretch level). Bottom: PI (left) and MI (right) maps immediately after the lesion and after reorganization. Each map displays labels on all neurons (left) or only the neurons that are maximally activated by that muscle. From Goodall et al. (1997), reprinted with permission.

In the work of Goodall et al. (1997), an existing computational model of the sensorimotor control of arm movements (Chen and Reggia, 1996), incorporating a model of both the somatosensory and the motor cortex, was used to investigate the reorganization processes that occur immediately after a focal cortical lesion; see Figure 1. The model assumes “mexican-hat” lateral cortical connections, a competitive activation dynamics and a Hebbian plasticity mechanism for incoming cortical connections. Lesions were modeled by setting the activation levels of selected units to zero, and by eliminating connections to and from those units.

The main prediction of this model is a two-phases reorganization process. Immediately after the lesion, lower activity is observed in the areas surrounding the lesion. A second phase is characterized by a gradual increase of the size of this area and by a general reorganization of the intact cortical regions. Both effects are mediated by activity-dependent synaptic changes. The low-activity peri-lesional area and its expansion over time are due to lack of activation. The model also predicts that a small uniform excitatory peri-lesional input may favor the participation of this area in the reorganization process.

The same group also studied the short- and long-term changes in lateralization that occur after a focal lesion, and investigated the possible contribution to recovery of the intact hemisphere and of inter-hemispheric communication (Reggia et al., 2000; Shkuro et al., 2000).

Here cortical reorganization is modeled in terms of synaptic changes (self-organization) of a topological map. In contrast, Butz et al. (2009) specifically addressed the mechanisms of activity-dependent synaptic rewiring that occur immediately after a focal cortical lesion. Synapse formation is accounted for by models of axonal and dendritic elements. On the pre-synaptic side, activity is assumed to promote axonal outgrowth. On the post-synaptic side, each neuron is assumed to change its input connectivity in an homeostatic manner, with the goal of keeping the firing probability within a specified range. In this framework, rewiring after a lesion can be seen as a form of compensation, driven by the need to regain a stable (homeostatic) regime.

The main prediction of this model is that neural populations that are already in homeostatic conditions (e.g., in adult individuals) are much less likely to compensate for lesions than networks that are still under development.

An additional prediction is that external stimulation may promote axonal outgrowth, thus accelerating rewiring. However, prolonged stimulation may induce a saturation effect, hence it can be detrimental to recovery or, anyway, less effective than paused stimulation. In terms of rehabilitation, these findings suggest that training with pauses in between may be more effective than continuous intensive training without pauses.

Varier et al. (2011) used the model proposed by Reggia and colleagues (Chen and Reggia, 1996) to examine the effects of focal and distributed lesions at various stages of development. In partial contrast with Butz et al. (2009), this model predicts that mature systems are relatively more robust to lesions than systems that are still under development. This apparent discrepancy may be a consequence of the different assumptions on structural plasticity—highly modifiable (Butz et al., 2009) vs hardwired (Chen and Reggia, 1996).

Reinkensmeyer et al. (2003) developed a model of cortical damage and its consequences on arm reaching movements. Different from the previous approaches, this model does not address intracortical connectivity and its reorganization. Based on experiments on non-human primates (Georgopoulos et al., 1982), neurons in the motor cortex are assumed to collectively encode the initial direction of the movement (population vector coding). Specifically, each neuron’s firing rate is assumed to be a function (truncated cosine) of the difference between the actual direction and the “preferred direction” for that neuron plus a noise term, whose standard deviation is proportional to the deterministic part of the cell response (signal-dependent noise). The overall encoded direction is the sum of the preferred directions of each individual neuron, weighted by their activity levels.

Cortical lesions were simulated by eliminating part of the neurons (cell death)—hence resulting in under-represented or non-represented preferred directions. Movement performance was measured in terms of the discrepancy between intended and encoded movement direction. They specifically looked at the variability of directional error within the same intended direction and across directions, and how these quantities are affected by cell death. They found that both types of error are inversely correlated with the fraction of surviving cells. In a number of experimental studies with stroke survivors, they found that the same indicators exhibit similar relationships with the subjects’ clinical impairment score (Kamper et al., 2002; Reinkensmeyer et al., 2002; Takahashi and Reinkensmeyer, 2003).

This study addresses the problem of how cortical damage results in impaired movements. As such, it is a model of impairment, which does not explicitly address the mechanisms of recovery.

### Models of use-dependent recovery

**Figure 2:**
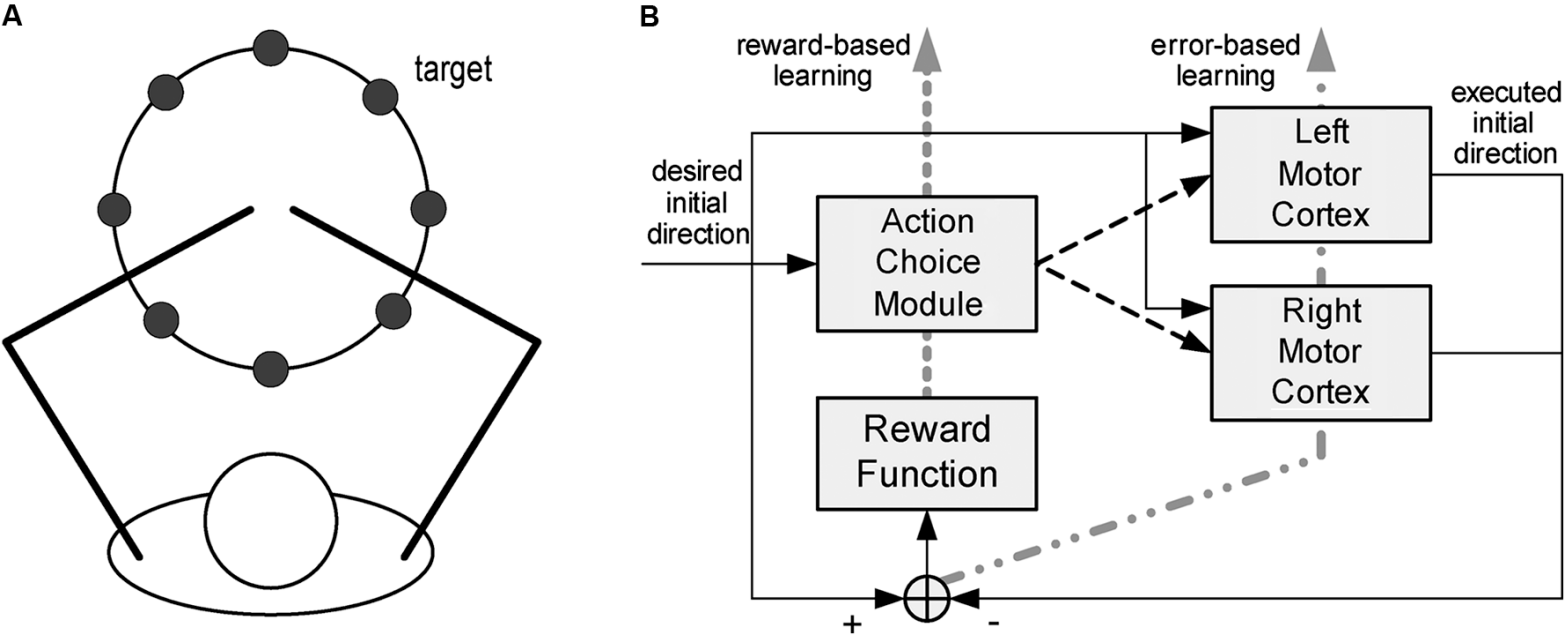
**Model of spontaneous recovery after stroke.** The model focuses on reaching movements **(A)**, for which subjects spontaneously select what arm to use. The model **(B)** consists of a model of the motor cortex (both hemispheres) and an action selection module. From Han et al. (2008), reprinted with permission.

Other models focus on cortical reorganization in the context of a specific motor task. Han et al. (2008) look at how lesions in cortical motor areas affect the mechanisms of arm selection to achieve a goal (reaching a target), and how impairment evolves through spontaneous arm use. The model accounts for motor cortical dynamics (both hemispheres) and action selection; see Figure 2. The cortex is modeled as in Reinkensmeyer et al. (2003), with the addition of a Hebbian learning mechanism to account for cortical reorganization. In addition, the process of deciding which arm to use is modeled as a form of reinforcement learning.

More specifically, the preferred directions for each neuron are assumed to adapt as a function of activity. Adaptation has two aims: (i) shifting the actual encoded direction closer to the desired direction (supervised component) and (ii) shifting the preferred directions of the individual neurons toward the desired direction (self-organizing component).

The action selection module accounts for the process of selecting the hand that will actually make the movement. A model of the action-value mapping, based on radial basis functions, generates the expected reward as a function of the direction of the actual movement. The hand whose expected reward is maximal is selected to execute the movement. This module is driven by a reinforcement learning mechanism. After every movement a reward signal is provided, defined as the sum of two terms, respectively reflecting (i) how close the cortex’s encoded direction is to the desired movement direction, and (ii) the fact that the left hand is more likely chosen for leftward movements whereas the right hand is more likely selected for rightward movements. The action-value model is updated to minimize the difference between actual and expected reward.

As in Reinkensmeyer et al. (2003), the effect of a stroke was modeled by eliminating part of the neurons within one hemisphere’s motor cortex. As a result, the impaired side is initially unlikely to be selected for movements on that side, and lack of use makes its selection even less likely. In contrast, its forced use induces reorganization, so that the intact portion of that hemisphere gradually shifts its preferred directions toward those that were once covered by the impaired portion of the cortex. In summary, the model addresses the mechanisms of interaction between activity-driven cortical reorganization and functional compensation, i.e., the change in the motor strategy (in this case, from the impaired to intact arm) that is driven by the need to preserve functional performance (e.g., a high reward).

Takiyama and Okada (2012) used a similar model, with emphasis on bimanual training. Their main prediction is that bimanual training facilitates the adaptation of the preferred directions of the unimpaired neurons in the ipsilesional cortical hemisiphere.

Han et al.’s (2008) model predicts that recovery will self-sustain if the amount of spontaneous use of the impaired arm reaches a certain threshold. If this is not the case, the impaired arm will be less likely to be selected, and recovery (if any) will gradually wash out. The model makes an important qualitative prediction—an activity threshold is a necessary condition for recovery to self-sustain. This can help explaining the mechanisms of action of rehabilitation strategies that rely on forced use of the impaired arm. As a matter of fact, observations from a rehabilitation trial based on constraint-induced movement therapy (CIMT) were found to be consistent with the “threshold” notion (Schweighofer et al., 2009). Aiming to achieve an “activity threshold” rather than providing a fixed amount of training, can be seen as a form of personalization of the therapy. Studies in this direction are currently under way, one first step being to quantify the amount of arm non-use in activities of daily living (Han et al., 2013).

More in general, the model is important because it is a first attempt to address the interplay between cortical reorganization and the development of compensatory strategies.

Although with different emphasis, all the above approaches focus on neural mechanisms of use-dependent reorganization. Impairment is only modeled qualitatively, and as such these models cannot immediately be related to the behavior of a specific subject, if not in qualitative terms.

**Figure 3:**
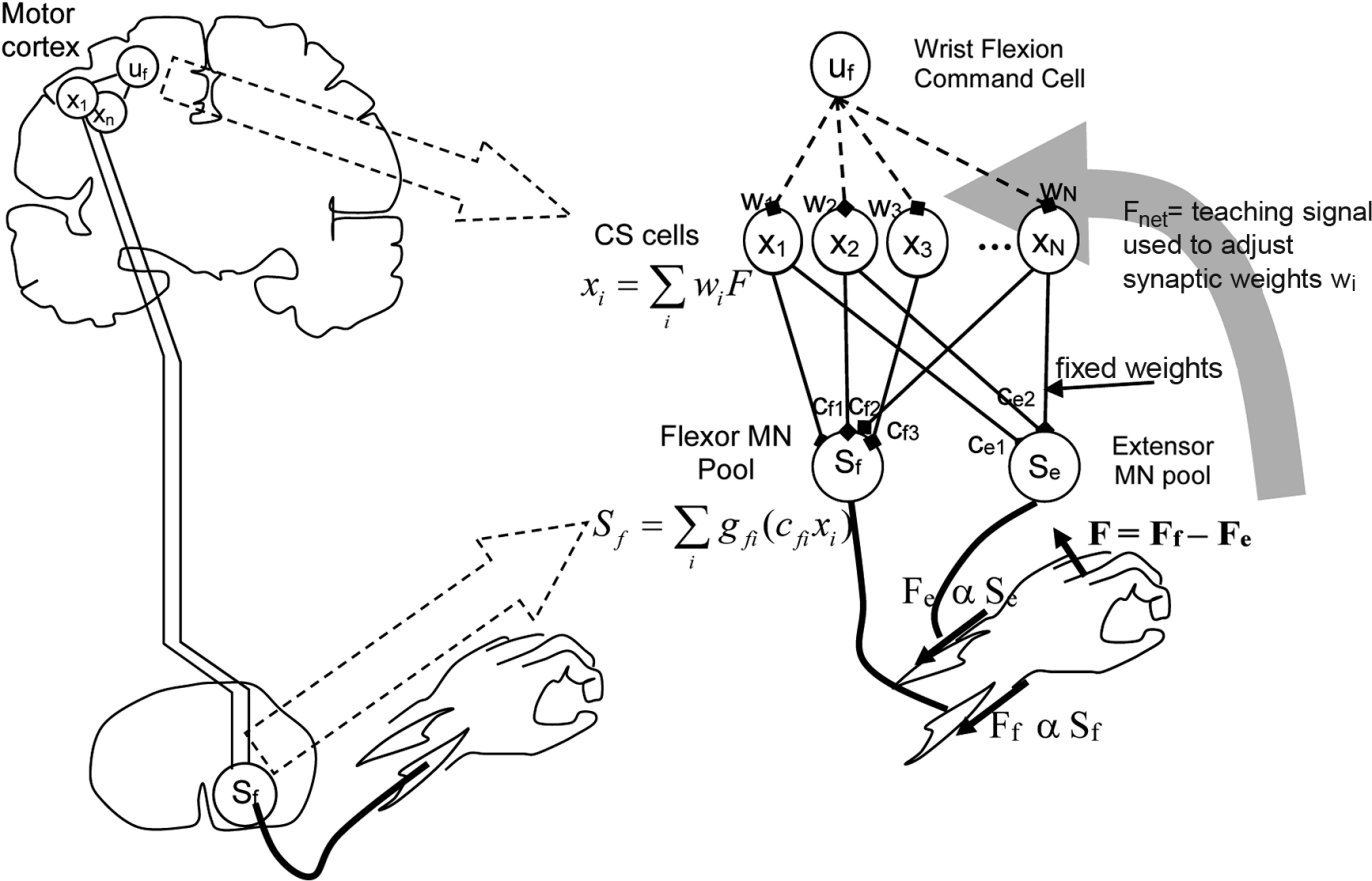
**Model of cortico-spinal reorganization after stroke**. From Reinkensmeyer et al. (2012a), reprinted with permission.

Two related studies (Reinkensmeyer et al., 2009b, 2012a) focus on how reorganization of the preserved cortico-spinal (CS) pathways and the recruitment of new ones underlie the recovery of the ability to generate force. The proposed model—inspired by single-cell recordings of the neural correlates of wrist force generation in primates—specifically addresses the “residual capacity” phenomenon, i.e., the empirical evidence that additional motor training may still improve the movement capacities even years after a stroke.

Specifically, the model is based on the notion that experience of movement “practice” induces a re-optimization in the recruitment of the intact CS connections to motor-neurons (MNs). A pool of CS cells (see Figure 3) is assumed to receive a movement command as input. The activity of CS cells summates at the level of the MN pools in the spinal cord. Wrist force (aimed toward either flexion or extension) is determined by the difference between the activities of the “flexor” and “extensor” MN pools. This can be considered as the simplest muscle synergy. The synaptic weights of the CS-MN connections are assumed to be fixed, whereas the weights of the input connections to the CS cells are learned through a reinforcement mechanism, in which the experienced attempt to move the limb represents the reward signal that guides the refinement of activation.

Furthermore, the model assumes that if CS connections originating from the primary motor cortex (M1) are lacking, CS connections from the supplementary motor area (SMA) may be recruited as well.

Model simulations predict that the size of the residual CS population determines the maximum strength an individual stroke patient can achieve. This is consistent with observations based on Transcranial Magnetic Stimulation (TMS) and Magnetic Resonance-Diffusion Tensor Imaging (MR-DTI) (Stinear et al., 2007) suggesting a strong correlation between white matter integrity and maximum grip force. In addition, the model predicts that the same dose of exercise is more effective when administered to sub-acute subjects (as compared to chronic), and to less impaired subjects (as compared to more severe). Furthermore, severe M1 lesions are predicted to induce an increase in SMA activity. These predictions have been confirmed experimentally (Feydy et al., 2002; Ward et al., 2007).

This model has not been directly used in rehabilitation, but provides some hints on how to make rehabilitation more effective. The simulations show that recovery would be facilitated if noise level were decreased, or if noise were signal-dependent. Another prediction is that inhibition of the stronger connections, e.g., the residual connections originating from M1, would facilitate the recruitment of alternative pathways. The latter prediction is somehow consistent with, and actually provides a possible interpretation for, the empirical observation (Bolognini et al., 2009) that repetitive TMS of the intact cortical hemisphere facilitates recovery.

Like Han et al. (2008), this model only makes qualitative predictions and cannot describe the behavior of one specific subject. Further, apart from the M1-SMA shift of activity, the model does not directly address the compensation issue and does not provide new insight about the interplay between neural reorganization and task re-learning.

### Temporal evolution of performance over training time

Variants of the power law of practice have often been used to describe the trial-by-trial evolution of motor performance during exercise, both within and between training sessions (the “functional” level of description).

In a widely used approach to robot-assisted rehabilitation (Krebs et al., 1998; Colombo et al., 2007), subjects are allowed a specific time interval to complete the task without assistance. After that, the robot completes the task through a high-stiffness position controller. In this way, the amount of robot intervention (fraction of the movement operated by the robot) automatically adjusts to subject’s performance (more active performance, less robot intervention). However, to prevent movements from being totally passive, at least initially, this technique requires at least a minimum residual amount of voluntary control. In a systematic analysis of various performance indicators during robot-assisted rehabilitation based on this protocol, Colombo et al. (2008, 2010) used an exponential model to account for their temporal evolution.

The main finding of these studies was that some movement features, namely force control and movement smoothness, improved more quickly than parameters that relate to the fine-tuning of the movement, like speed. Specifically, speed improvement exhibited a much longer (2–3 times) time constant than force control and smoothness. Furthermore, in several subjects the path curvature exhibited a non-monotonic time course, with an initial increase until a peak, followed by a steady decrease.

These findings shed some light on the process of functional recovery. Subjects first explore the action space with the primary objective of exploiting their residual abilities to achieve the goal; then they undergo effort optimization to make the movement more efficient. The distinctive behavior of path curvature is a consequence of this two-phase process: the increase relates to the exploration phase, whereas the decay denotes the beginning of the effort optimization phase. The final step is the fine tuning of movement performance, denoted by the increase in movement speed (Colombo et al., 2012). These processes likely start at the same time and run in parallel, but have different time constants (faster the former, slower the latter).

A different, but related view of recovery is based on the notion that each movement is built by combining multiple sub-movements, and the empirical observation (Dipietro et al., 2009) that neuromotor recovery is characterized by a gradual decrease in the number of sub-movements—which results in an improved smoothness.

These mechanisms suggest that training-mediated recovery shares common features with motor skill learning.

In a subsequent study, Colombo et al. (2012) developed a control algorithm Progressive Task Regulation (PTR) that automatically adjusts various aspects of the task (amplitude of the movement, number of subtasks, assistance modality) according to the evolution of the different performance indicators. After each training epoch, performance is measured (moving average over the last three epochs), and the difficulty level of the task is modified according to a set of rules. Depending on the fraction of movement completed without assistance of the robot, exercise difficulty can switch to more challenging assistance modalities. The observed evolution of the different performance indicators was incorporated into the threshold values used to determine when the switch occurs. The decisions of switching task difficulty based on the PTR algorithm were found to be highly consistent with those based on subjective therapists’ assessment.

This model only focuses on performance time series and only provides information on the temporal evolution of the recovery process. However, it does not attempt to describe the mechanisms underlying recovery. Specifically, the model describes the evolution of performance in non-assisted trials. The same modeling framework can address situations in which the degree of assistance is constant or changes slowly and monotonically, but cannot distinguish between the “robot” and the actual “voluntary” contributions in situations when they act together.

Recently, the same group proposed a dynamical model of recovery (Panarese et al., 2012), attempting to reproduce the mechanisms underlying the trial-by-trial evolution of performance. The model derives from a computational framework that was originally developed by Smith et al. (2004) in the context of associative learning in animal studies. In this model, an internal state variable denoting “motor improvement” is modeled as a random walk. This reflects the assumption that motor improvement builds up as the effect of many different factors. As an addition to the original model, at a given trial the logarithms of a number of observable performance indicators are assumed to be proportional to the amount of motor improvement. The model was found to reproduce the variety of time constants of the recovery observed in the different performance indicators. While interesting, this model is nothing more than a noisy version of the exponential model. In particular, the random walk assumption says little on what determines recovery.

### The role of assistance

A few modeling studies have addressed the issue of how robot assistance affects recovery. Reinkensmeyer (2003) and Emken et al. (2007) focuses on adaptive changes in gait movements in presence of assistive forces. The study specifically addresses the trial-by-trial evolution of performance during adaptation to an assistive force field, and suggests that adaptation can be explained as an optimization process, which accounts for a combination of motor error and effort.

The resulting computational model is summarized in Figure 4. Similar to force field adaptation experiments (Thoroughman and Shadmehr, 2000; Scheidt et al., 2001; Donchin et al., 2003; Cheng and Sabes, 2006), the adaptation process is modeled as a linear autoregressive model. A controller receives as inputs the desired trajectory, the performance error and the motor command (muscle force) on the previous trial. The motor command on the next trial is the controller output. The controller includes two modules: (i) an inverse model of limb dynamics, which transforms a desired trajectory into an appropriate motor command; (ii) a “learning rule”, which adapts the motor command to changes in the dynamics of the limb and/or of the environment. The output muscle force is applied to the body, whose movements are disturbed by an external perturbation.

**F4:**
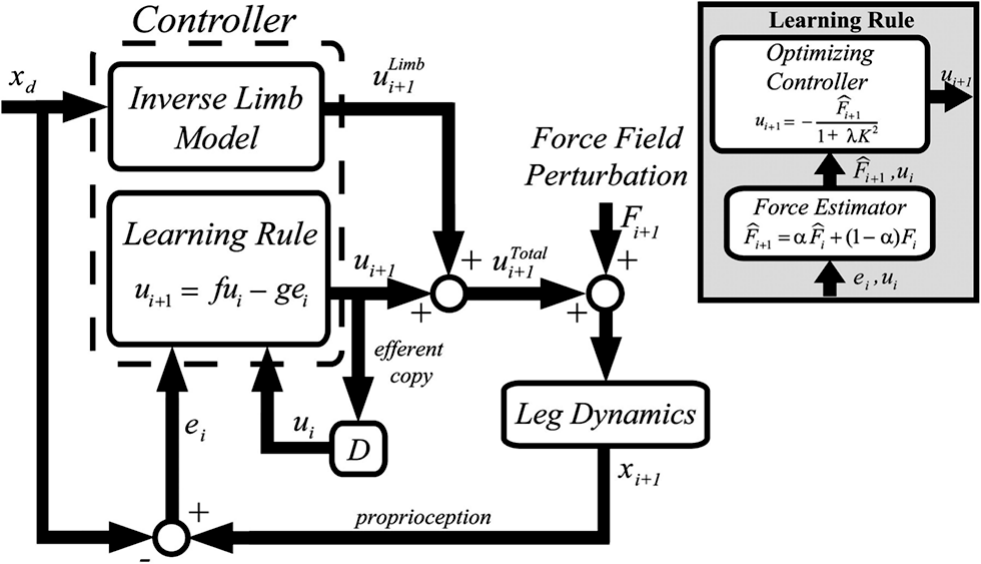
**Greedy optimizer model.** The controller includes an inverse model of the limb and a “learning rule”. The latter is based on a force estimator and an optimal controller. From Emken et al. (2007), reprinted with permission.

The “learning rule” module (Figure 4, inset) relies on the notion that while dealing with assistive forces the motor system behaves as a “greedy” optimizer, so that these forces are quickly incorporated into the motor plan in order to minimize the effort while maintaining the required performance level (e.g., a small error). The force estimator predicts the disturbance on the next trial, and an optimizing controller generates the next motor command by minimizing a cost function on a trial-by-trial basis. In the model derived from this optimization framework, the “slacking” mechanism is captured by a decay term in the dynamics equation (the “f” term in the Learning Rule block, see Figure 4).

Emken et al. (2007) also suggested that slacking may have adverse effects on recovery. As a consequence, in robot-assisted rehabilitation, assistance should be kept to a minimum. Furthermore, it has to be decreased—manually or automatically—as performance improves. A variety of techniques have been proposed to adaptively regulate the magnitude of assistive force as a function of the observed outcome. In Casadio et al. (2009) the therapist manually selected the assistance level in order to keep it to the minimum level that evoked a functional response needed for accomplishing the task. In Vergaro et al. (2010) a linear controller continuously and automatically regulated the assistive force provided by the robot, depending on on-line performance measures. Similar mechanisms have been proposed in the context of upper limb—the Performance-based progressive robot-assisted therapy used by the MIT-Manus robot (Krebs et al., 2003)—and the lower limb—the patient-cooperative training modality used by the Lokomat system (Riener et al., 2005; Mihelj et al., 2007). Using a computational model of human-robot load sharing, Reinkensmeyer et al. (2007) suggested that to achieve assistance as needed, the robot controller should possess a slacking mechanism that resembles that observed in humans. Wolbrecht et al. (2008) designed a more complex adaptive control scheme, based on task performance, that automatically negotiates an error-reducing and an effort-enhancing component. The controller needs an explicit model of the subject’s arm and its neural control. In this specific study, this model took the form of a radial basis function neural network, which was built by experience.

The “greedy optimizer” model has been highly influential to rehabilitation, but does not really address the recovery mechanisms. In fact, it has been derived from studies on healthy subjects and its implications for recovery are largely speculative. The slacking hypothesis has never been directly tested in clinical rehabilitation trials.

In a recent study, Casadio and Sanguineti (2012) developed a linear dynamical model to describe the trial-by-trial evolution of the motor performance of chronic stroke survivors who underwent a rehabilitation protocol based on a robot-assisted arm extension task.

**Figure 5:**
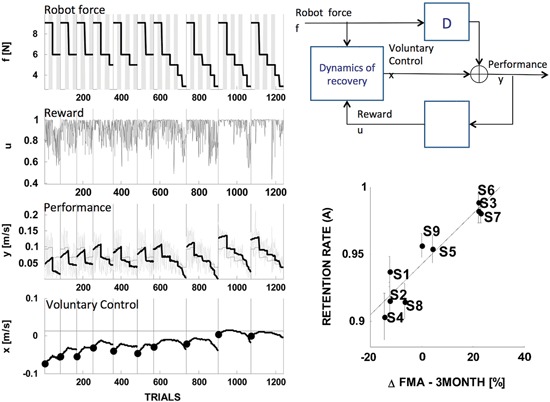
**State-space model of stroke recovery.** Left: fitting performance. Right: model schematic (top) and the main result, that the model’s rate of retention parameter predicts the long-term outcome— change in the Fugl-Meyer score (FMA) in the three months following the end of the treatment (bottom). Modified from Casadio and Sanguineti (2012).

The model is based on the notion that in robot-assisted exercise the robot device and the subject cooperate toward a common goal—a form of shared control. Specifically, the model assumes that task performance is a function of a voluntary, human-generated command (taken as the model’s state variable) and of a robot-generated assistive force (taken as one of the model’s inputs); see Figure 5, top right.

As regards the dynamics of the actual recovery process, the model assumes that the amount of voluntary control on the next trial is the sum of three components: (i) a “memory” or “retention” term—a fraction of the current amount of voluntary control; (ii) a “learning” component, proportional to a “driving” signal—a function of movement performance that can be interpreted as a reward—and (iii) an assistance-related component—proportional to the magnitude of the assistive force.

A distinctive feature of this model is that it posits separate mechanisms for “retention” and for the effect of assistance, i.e., the actual “slacking”. These terms have often been used interchangeably; see Reinkensmeyer et al. (2009a) for a review that specifically covers slacking models.

The model was used to analyze the trial-by-trial time series of performance in nine chronic stroke survivors, who underwent a 10-sessions training protocol; see Figure 5, left. The estimates of the model parameters for each subject suggested that recovery is determined by a complex interplay of memory (retention), performance and slacking. One specific finding was that in severely impaired subjects recovery is greater when the driving (reward) signal is greater; hence, recovery improves when the performance—not the motor error—is greater. Another finding, which somewhat confirmed the observation of Emken et al. (2007), was that a greater assistive force has a negative impact on recovery (slacking). However, only a few subjects—the least impaired—exhibited a significant “slacking” effect. The single most important finding was that the retention rate (memory decay) parameter accurately predicts the long-term outcome of the rehabilitation trial (see Figure 5, bottom right). This finding is consistent with Han et al. (2008): the hypothesis that recovery must reach a threshold in order to self-sustain implies a buildup mechanism that integrates the effect of repeated motor activities. High retention is an essential prerequisite of this mechanism.

This model suggests that mechanisms of recovery may differ in different subjects. Again, this calls for an adaptive regulation of assistance, in which peculiarities of the individual subjects are to be taken into account.

Squeri et al. (2011) designed an adaptive Bayesian regulator that adjusts the magnitude of assistive force (or other task parameter) to keep the average performance around a target magnitude (performance clamp). In this way, as performance improved, the controller automatically reduced the amount of assistance. Squeri et al. (2012) used this model to analyze the temporal evolution of the subjects’ voluntary control in a task that involved sub-movements in different directions. One single controller was used to adaptively regulate the degree of assistance in all sub-movements. The model suggested that the dynamics of recovery is direction-dependent, mostly due to the different sensitivity to assistance exhibited by the arm moving in different directions. These results suggest that modulation of assistance would better be made separately for each direction. More in general, they point at the need for therapy personalization.

While this model attempts to distinguish between recovery and slacking (i.e., adaptation to assistance), it remains purely descriptive and does not explicitly address the underlying mechanisms.

### Synergy-based models of impairment

The characterization of compensatory strategies in stroke survivors is receiving an increasing attention. A number of studies look at movements in terms of their basic building blocks, or synergies (Ting and Macpherson, 2005; Tresch et al., 2006; Raghavan et al., 2010; Cheung et al., 2012). The notion of muscle synergy relies on the assumption that the nervous system recruits spinal inter-neural modules that activate groups of muscles as individual units.

Cortical lesions affect the organization of these modules, thus resulting in abnormal muscle activations (Twitchell, 1951; Brunnstrom, 1970), incorrect regulation of interaction torques, incorrect timing of action sequences (Archambault et al., 1999), decreased joint range of motion (Levin and Dimov, 1997), loss of inter-joint coordination (Levin, 1996a) and ultimately abnormal movements

To extract muscle synergies, Cheung et al. (2012) collected myoelectric signals (EMGs) from different muscles and applied a factorization algorithm—Non-negative Matrix Factorization (NMF)—to the rectified, integrated, and variance-normalized EMGs. Based on this factorization, the temporal pattern of activation of each muscle is expressed as the sum of a small number of (time-varying, task-dependent) signals. The relative contribution of each of these signals to each muscle is time-invariant and task-independent, and denotes groups of muscles that are recruited together.

These authors systematically analyzed the muscle synergies exhibited by stroke survivors with different levels of impairment (mild to severe) and different disease durations (post-acute to super-chronic). Subjects performed different tasks with each of their arms to allow a comparison of their muscle synergies in both the impaired and the unimpaired sides. A portion of the synergies observed in the impaired arm was found to be similar to those found in the intact arm of the same subjects (“preserved” synergies). The remaining synergies were altered. Specifically, the authors identified two distinct types of alterations, which they named “merging” and “fractionation”. “Merging” refers to a situation in which in the impaired arm multiple synergies merge together. The amount of merging was found to correlate with the severity of the impairment. “Fractionation” refers to a situation in which the normal synergies split into multiple, novel patterns. The amount of fractionation was found to correlate with the disease duration (time since stroke onset), with suggest that it can be seen as a form of spontaneous reorganization.

Synergy models do not explicitly address the recovery process, but can be used to characterize impairment and the effects of treatment in individual subjects, in term of their repertoire of movement strategies at the articular and/or muscular level. In other words, muscle synergies are physiological markers of both the degree of impairment and of the degree of recovery. Dipietro et al. (2007) observed that in chronic stroke survivors, robot-assisted exercise results in a more efficient control of shoulder and elbow joints. They suggested that these changes are due to a “tuning” of the existing abnormal synergies, not to their modification.

Latash and Anson (1996) suggested that in impaired individuals, the modified motor strategies should not be necessarily considered as pathological but, rather, they should be seen as forms of adaptation to the primary disorder. Therefore, their correction should not be the primary concern of the rehabilitative treatment. However, this view may be in contrast with the long-term goal of recovering motor functions (Levin, 1996b; Cirstea and Levin, 2000). Failure to modify the abnormal synergies may lead to incorrect postures, weakening of underutilized muscles. With time, it may worsen the chances of recovering other abilities (Levin, 1996b).

It is conceivable to design technological aids that drive the recovery toward preserving and/or facilitating the “normal” articular and muscle synergies while, conversely, reducing or preventing the “abnormal” ones. Recently, Crocher et al. (2012) demonstrated that in healthy subjects, training with an exoskeleton may induce changes in the arm-related synergies. In severe stroke survivors Ellis et al. (2005) demonstrated that training reduces abnormal isometric elbow and shoulder joint torque coupling.

Rehabilitation treatments built upon synergy models should be designed to emphasize the synergies that are silent or under-activated, while discouraging compensatory strategies in favor of new and more independent coordination patterns.

Synergy-based models are descriptors of impairment; they do not provide an explicit model of the recovery process, although they can be used for describing this process as a change at the level of muscle activity.

## Discussion

Computational models are widely used to investigate motor skill learning and sensorimotor adaptation. Similar models might potentially contribute to our understanding of the mechanisms of recovery after a stroke at “functional” level, and to the design of optimal, individualized rehabilitation strategies.

Neuromotor recovery is facilitated by exercise and is mediated by neural reorganization at cortical and sub-cortical levels, whose physiological substrate is synaptic plasticity and rewiring through axonal outgrowth.

On one hand, neural plasticity mechanisms constrain the way recovery proceeds. On the other hand, sensorimotor behaviors determine the patterns of neural activity, thus inducing specific, activity-dependent synaptic changes. Therefore, behavioral mechanisms and neural reorganization cannot be simply treated as different levels of description. Rather, both aspects need to be accounted for when modeling the neuromotor recovery process.

### Do computational models provide testable hypotheses on the mechanisms of recovery?

It has been suggested (Krakauer, 2006; Dipietro et al., 2012) that neuromotor recovery shares at least some features with motor learning. The class of models focusing on the recovery of functions highlights this “motor learning” component.

To acquire (or re-acquire) a motor skill an individual must understand how to achieve the movement goal and, more in general, learn how to get high rewards, while minimizing the necessary muscle effort. Some of the models discussed in the previous sections unveil specific aspects of this process. The observation (Colombo et al., 2010) that different aspects of motor performance exhibit a different temporal evolution, which may not be monotonic, reflects the notion that recovery is a complex multifactorial process, in which maximization of performance is only one of the components. Another aspect is generalization, from simpler to more complex movements (Dipietro et al., 2009).

Other models (Han et al., 2008; Reinkensmeyer et al., 2012a) describe neuromotor recovery within a reinforcement learning paradigm. Casadio and Sanguineti (2012) suggest that in severe chronic stroke survivors, improvements in voluntary control are determined by performance, not error—another indication that recovery has much in common with motor skill learning.

Other models focus on the “central” level, and describe cortical reorganization in terms of Hebbian and/or self-organization principles. These models highlight a number of physiological mechanisms of recovery. The model of Goodall et al. (1997) predicts an initial increase of the size of peri-lesional “silent” areas immediately after the lesion, followed by overall reorganization. Another specific prediction is that tonic stimulation of the lesioned side would limit the size of peri-lesional “silent” areas. A similar effect is obtained by limiting the activity of the contralateral hemisphere. Butz et al. (2009) focuses on axonal outgrowth driven by the push toward restoring the homeostatic inter-hemispheric balance. Its main prediction is that stimulation may facilitate axonal outgrowth but, because of a saturation effect, paused stimulation is more effective than sustained stimulation.

Still other models address reorganization mechanisms either at the level of the musculoskeletal system or at the level of CS circuitry. Reinkensmeyer et al.’s (2012a) model includes a basic form of reorganization, based on the recruitment of corticospinal pathways that originate from cortical areas (e.g., the SMA) that in the intact brain are normally not used because they are less efficient, and therefore more “costly”, in contributing to force generation.

In Han et al.’s (2008) model, the activity of the impaired limb induces a reorganization of the ipsilesional cortical areas, which in turn makes this same hand more likely to be selected for movement. Its main prediction is that for recovery to self-sustain, activity of the impaired hand must reach a threshold. The hand selection (Han et al., 2008) model suggests that if there is too much emphasis on performing the task but there is too little pressure toward reorganization—for instance, if the affected arm is not likely to be selected and hence the impaired hemisphere is not active enough to undergo reorganization—recovery of the paretic side functions will not self-sustain and will possibly wash-out. One testable prediction is that techniques that facilitate reorganization independent of motor learning—e.g., by increasing cortical excitability, as with trans-cranial direct current stimulation (tDCS) (Reis et al., 2009) might lower the “threshol” activity level that allows recovery to self-sustain. The model of Takiyama and Okada (2012) is very similar. It predicts that the inter-hemispheric activation induced by bimanual exercise facilitates cortical reorganization on the ipsilesional side.

A complementary view of the recovery process is provided by the attempts to characterize muscle synergies in a quantitative way (Ting and Macpherson, 2005; Tresch et al., 2006; Raghavan et al., 2010; Cheung et al., 2012). These models do not directly address the recovery mechanisms, but provide a window into the way muscle groups are recruited in different phases of the recovery process, and allow to distinguish whether recovery occurs through structural changes in muscle synergies, or as a tuning of existing synergies.

Only few models address the facilitatory role of assistive forces. In principle, assistive forces enable achieving the same motor performance with less voluntary contribution (Emken et al., 2007), but how this mechanism plays a role in the recovery process has not been extensively investigated. The “slacking” hypothesis predicts that reduced voluntary commands have a detrimental effect on recovery. A recent study confirmed this prediction, by demonstrating that subjects undergoing passive training exhibit less recovery than subjects that are actively involved in the exercise through an electromyography-driven robot. Hu et al. (2009) and Casadio and Sanguineti (2012) found that this effect may not be equally important in all subjects.

### Can computational models predict the recovery on a patient-by-patient basis?

By “predicting the recovery” we mean estimating the future evolution—either spontaneous or induced by treatment—of the impairment and/or the functional performance of a specific subject, measured in terms of clinical scales and task-specific performance indicators. A number of mathematical models, e.g., Chaudhuri et al. (1988), Saeki et al. (1994), Oczkowski and Barreca (1997), Stineman et al. (1997), Lofgren et al. (2000) and Mirbagheri et al. (2012), have been proposed to predict the recovery outcome within different time scales. These models use different types of information, such as the initial degree of impairment and the nature, size or location of the lesion. In contrast, computational models explicitly account for the mechanisms by which recovery takes place, namely use-dependent neural reorganization and motor learning.

Some computational models focus on general principles, but do not address the dynamics of the recovery process of a specific individual. All models of the central level account for brain areas, CS circuitry and neural plasticity mechanisms in a way that cannot be immediately associated to empirical observations on individual subjects.

However, some comparison of these models with empirical data is still possible. For instance, in Reinkensmeyer et al. (2003) the lesion is modeled as a reduction in the number of available direction-tuned cells in the motor map. The size of the lesion can be related to the degree of impairment, a correlation that has been observed empirically. In principle it would be possible to personalize the model in terms of location and size of the lesion, or fraction of intact CS pathways (e.g., from imaging data), but the general focus is on quantities (e.g., the activity and the changes of the spatial tuning individual cortical columns) that are hard to associate to empirical measures.

Other models allow inferring how recovery would take place in a specific individual. These include all functional models and synergy-based models.

Specifically, Colombo et al. (2010) predict the time constant of the recovery process by fitting the time course of the performance data during the rehabilitation treatment. Casadio and Sanguineti (2012) predict the long-term retention of the recovery by using a state space model and by looking at the memory decay of the learning process. These models directly refer to observable quantities, so that it is relatively easy to identify subject-specific model parameters. Model parameters capture the modality with which one subject undergoes recovery. For instance, a model may allow estimating the subject’s rate of spontaneous recovery, or may provide information on his/her peculiar response to mechanical perturbations (mechanical impedance).

All the above models enable significant, but limited predictions. All focus on limited aspects of the recovery process (cortical, muscular, functional) and only provide a limited account of their complex interplay. The importance of such interplay is exemplified by the prediction that the possibility that recovery will self-sustain can be inferred by evaluating if the amount of spontaneous use of the impaired arm reaches a certain threshold (Han et al., 2008).

In conclusion, multiple levels of description would be necessary. One important issue is whether these models can incorporate the specific features of one patient (nature, size and location of the lesion; type and degree of impairment). This is particularly important for the cortical level, for which there is a need for descriptions that are based on observable quantities.

### Do models allow designing patient-specific “optimal” therapy?

Computational models may enable designing patient-specific therapy, aimed at maximizing speed and amount of recovery of that patient.

Patient-specific models provide a description of his/her status and characteristics, e.g., impairment, articular and muscular synergies, residual movements and force generation abilities etc. They also provide a better understanding of what determines the recovery of that patient. This information can be used to define specific goals for treatment, and to assess its efficacy in terms of progress of the subject’s status (motor strategies and functional behavior) toward the treatment goals.

Colombo et al. (2012) designed and tested a controller PTR that automatically selects exercise parameters (amplitude of the movement, number of sub-movements, assistance modality) based on their previous work on modeling the evolution of different performance indicators (Colombo et al., 2010).

Reinkensmeyer et al. (2007) proposed a robot controller based on the assist-as-needed principle, with a slacking mechanism similar to the one observed in humans. In several other applications, the magnitude of assistive force is adaptively regulated as a function of the observed outcome, in both the upper limb (Krebs et al., 2003; Vergaro et al., 2010) and the lower limb (Riener et al., 2005; Mihelj et al., 2007).

Bayesian regulation of robot-generated assistance (Squeri et al., 2011) is a direct derivation of the model of recovery proposed by Casadio and Sanguineti (2012).

The success of the above mentioned approaches suggests that an even greater advantage would come from the much more ambitious goal of designing treatment modalities that directly rely on patient-specific recovery models. In principle, if a model allows predicting evolution and final outcome of a specific rehabilitation intervention, it should be possible to use this same model as a basis for an optimal planning of the intervention. This may include the timing of the individual exercise sessions, the specific exercises to be administered, the trial-by-trial regulation of the degree of assistance, and the online planning of assistive or resistive forces (or other forms of stimulation, like Functional Electrical Stimulation (FES)).

Only few examples of model-based robot controllers for treatment have been proposed so far (Wolbrecht et al., 2008; Reinkensmeyer et al., 2012b). One major difficulty is that to incorporate recovery models in the robot controller requires to achieve a dual goal: the controller should select the goal and the difficulty level of the exercise based on the subject’s state as predicted by the model and, at the same time, the model should be continuously adjusted, on the basis of the observed subject and robot performance while the treatment proceeds. Therefore, the resulting treatment protocol has to be a trade-off between exploitation (of the model) and exploration (of the treatment control space to keep the model up to date).

## Directions for future research

Computational approaches to the study of neuromotor recovery after stroke are innovative and promising, but still in their infancy. The models described in this review open a new view of the recovery process, and along these lines there is still a lot to understand, to discover, and to integrate in the clinical practice.

We can identify the following directions for future research: (i) multi-level models; (ii) the role of modularity in neuromotor recovery, and (iii) new modeling approaches.

The models reviewed in this paper capture important and complementary aspects of the recovery process at central, muscular, functional level. With few exceptions, the focus is on one single level of description. Convergence toward multiple levels of description may provide a more comprehensive representation of the different aspects of the recovery process and of their interconnections. Multi-level models are being successfully used in other related fields such as musculo-skeletal disorders—see Fregly et al. (2012) for review—and are of great importance both for a more comprehensive understanding of the mechanisms related to the observed phenomenon—in our case, neuromotor recovery—and for planning the most appropriate intervention.

The well-established notion of modularity in the motor system has been getting a renewed attention in computational motor control. In the context of neuromotor recovery, the view of motor strategies as the combination of a repertoire of muscle synergies may be the key to unveil the role of mechanical redundancy in counteracting the effects of a central focal lesion, either toward compensation or true recovery. A recent study (Overduin et al., 2012) highlighted the neural correlates of muscle synergies in two rhesus macaque monkeys. However, more work is needed to understand how a focal lesion affects muscle synergies and how they evolve over time as a consequence of spontaneous recovery and/or exercise-based therapy.

One main difficulty in this application domain is our limited understanding of the physiological mechanisms underlying synaptic plasticity and neural reorganization. Moreover, these phenomena are hard to monitor and quantify experimentally, so that models at central level are difficult to identify from empirical observations and have poor predictive power. Novel modeling approaches, relying on quantities that can be observed with simple and non-invasive procedures, like fMRI and EEG would facilitate progress in this respect. Novel modeling approaches can also be beneficial to the study of the functional level of description. Novel computational approaches used for describing the neural control of movements and motor learning might find insightful application in the study of the recovery process. Optimization is a particularly viable concept to describe neuromotor recovery. Given the available sensory information and the constraints that derive from the actual impairment (sensory, motor), it may suggest how the next voluntary command is selected. Bayesian inference, optimal control and reinforcement learning may play a role here.

Computational models have been successfully applied to the study of motor learning and adaptation, providing important insights with respect how brain controls movement and react to the environment or task variables changes. Their application to the rehabilitation field is fairly new and the first approaches suggest that they will lead to a deeper understanding of the mechanisms underlying neuromotor recovery.

## Conflict of interest statement

The authors declare that the research was conducted in the absence of any commercial or financial relationships that could be construed as a potential conflict of interest.
